# Efficacy and Mechanism of Angiotensin II Receptor Blocker Treatment in Experimental Abdominal Aortic Aneurysms

**DOI:** 10.1371/journal.pone.0049642

**Published:** 2012-12-03

**Authors:** Yasunori Iida, Baohui Xu, Geoffrey M. Schultz, Vinca Chow, Julie J. White, Shola Sulaimon, Ayala Hezi-Yamit, Susan Rea Peterson, Ronald L. Dalman

**Affiliations:** 1 Division of Vascular Surgery, Department of Surgery, Stanford University School of Medicine, Stanford, California, United States of America; 2 Medtronic Vascular Inc., Santa Rosa, California, United States of America; University Medical Center Utrecht, The Netherlands

## Abstract

**Background:**

Despite the importance of the renin-angiotensin (Ang) system in abdominal aortic aneurysm (AAA) pathogenesis, strategies targeting this system to prevent clinical aneurysm progression remain controversial and unproven. We compared the relative efficacy of two Ang II type 1 receptor blockers, telmisartan and irbesartan, in limiting experimental AAAs in distinct mouse models of aneurysm disease.

**Methodology/Principal Findings:**

AAAs were induced using either 1) Ang II subcutaneous infusion (1000 ng/kg/min) for 28 days in male ApoE^−/−^ mice, or 2) transient intra-aortic porcine pancreatic elastase infusion in male C57BL/6 mice. One week prior to AAA creation, mice started to daily receive irbesartan (50 mg/kg), telmisartan (10 mg/kg), fluvastatin (40 mg/kg), bosentan (100 mg/kg), doxycycline (100 mg/kg) or vehicle alone. Efficacy was determined via serial *in vivo* aortic diameter measurements, histopathology and gene expression analysis at sacrifice. Aortic aneurysms developed in 67% of Ang II-infused ApoE^−/−^ mice fed with standard chow and water alone (n = 15), and 40% died of rupture. Strikingly, no telmisartan-treated mouse developed an AAA (n = 14). Both telmisartan and irbesartan limited aneurysm enlargement, medial elastolysis, smooth muscle attenuation, macrophage infiltration, adventitial neocapillary formation, and the expression of proteinases and proinflammatory mediators. Doxycycline, fluvastatin and bosentan did not influence aneurysm progression. Telmisartan was also highly effective in intra-aortic porcine pancreatic elastase infusion-induced AAAs, a second AAA model that did not require exogenous Ang II infusion.

**Conclusion/Significance:**

Telmisartan suppresses experimental aneurysms in a model-independent manner and may prove valuable in limiting clinical disease progression.

## Introduction

Abdominal aortic aneurysm (AAA) is an age-related, life-threatening degenerative vascular condition. Pathologic hallmarks include transmural aortic leukocyte infiltration, neocapillary formation or angiogenesis, progressive medial elastolysis and smooth muscle cell (SMC) depletion [1]. Although investigations using both human and experimental aneurysmal tissues have provided significant insights into AAA pathogenesis [1], [2], to date no pharmacologic strategy has proven effective in limiting aneurysm progression or reducing risk of rupture [3], [4].

Clinical and experimental evidence suggests that the renin-angiotensin (Ang) system (RAS), a critical blood pressure regulation mechanism, plays a significant role in AAA pathogenesis [5], [6]. For example, the protein expression levels of angiotensinogen and Ang II type 1 receptor (AT1) are elevated in human AAAs as compared to healthy and atherosclerotic aortae [7]. In hyperlipidemic mice, exogenous Ang II accelerates aneurysm progression [8], [9], whereas global or endothelial cell-specific deletion of AT1a attenuates AAA development [10], [11]. Ang converting enzyme (ACE) inhibitors consistently limit experimental AAA progression [12], but the efficacy of AT1 blockers (ARBs) varies by animal models and ARB compounds tested [12]–[15]. The efficacy of both ACE inhibitors and ARBs in limiting clinical AAA progression remains uncertain [16]–[18]. In the search for effective pharmacologic strategies to limit AAA progression, further investigation into the role of RAS targeting is warranted.

In this study, two ARBs, telmisartan and irbesartan, were compared to doxycycline, fluvastatin and the endothelin-1 receptor blocker bosentan for their ability to limit experimental aneurysm progression. Telmisartan and irbesartan were chosen because of their higher bioavailability and longer half-life *in vivo* than other ARBs [19]. Doxycycline, a pan-matrix metalloproteinase (MMP) inhibitor, has proven generally effective in limiting experimental aneurysm progression, albeit in a model-dependant manner [20]–[24], and has been trialed previously for early AAA disease suppression [25], [26]. Fluvastatin was chosen as a representative HMG-CoA reductase inhibitor (statin) for comparison to the ARBs and doxycycline [27]–[30]. Bosentan, an endothelin 1 receptor blocker, was included to isolate the relative influence of blood-pressure modulation on Ang II-induced experimental aneurysm progression. The goal of these experiments was to address previously conflicting data on the inhibitory efficacy of ARBs in experimental aneurysms, and to provide guidance on candidate agent selection for a clinical trial of pharmacologic suppression of early AAA disease.

## Results

### ARBs Suppress AAA Incidence and Reduce AAA-associated Mortality

**Figure 1:**
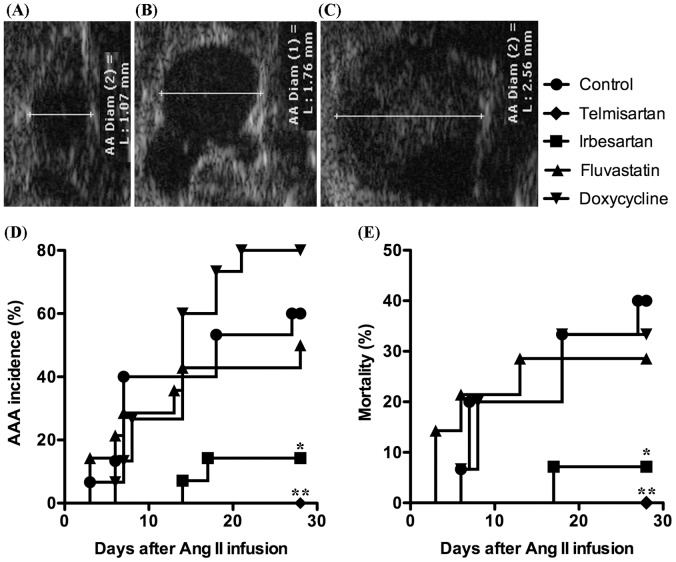
Influence of drug treatment on AAA incidence and mortality. **A–C:** Ultrasound images representing an intact aorta (A), an aneurysmal aorta without dissection (B) and an aneurysmal aorta with medial dissection (C). **D, E:** AAA incidence (D) and mortality (E) in Ang II-infused mice treated with telmisartan, irbesartan, fluvastatin or doxycycline. Kaplan-Meier analysis, *P<0.05 and **P<0.01 compared to control group, n = 14–15 mice in each group.

We monitored the suprarenal aorta in apolipoprotein E deficient (ApoE^−/−^) male mice during continuous subcutaneous Ang II (1000 ng/kg/min) infusion for 28 days with transabdominal ultrasound at 40 MHz. In this model (Ang II/ApoE^−/−^), an AAA was defined as a ≥50% increase in aortic diameter or the onset of dissection. Sixty-seven percent (10/15) of Ang II-infused ApoE^−/−^ mice fed standard chow and water developed AAAs within 28 days (Fig. 1D). In contrast, only 7% (1/14) of Ang II-infused ApoE^−/−^ mice treated with irbesartan (50 mg/kg/d in chow) developed AAAs, and none (0/14) of mice treated with telmisartan (10 mg/kg/d in chow) developed an AAA (Fig. 1D). Treatment with both ARBs also significantly reduced AAA-associated mortality compared to mice fed standard chow (Fig. 1E). By comparison, neither fluvastatin (40 mg/kg/d in chow) nor doxycycline (100 mg/kg/d in drinking water) treatment significantly influenced AAA incidence or mortality (Figs. 1D & 1E). Thus, telmisartan and irbesartan, but not doxycycline and fluvastatin, are highly effective at reducing AAA incidence and mortality in the Ang II/ApoE^−/−^ model.

### ARBs Attenuate Aortic Aaneurysm Progression during Chronic Ang II Infusion

Consistent with the impact on incidence and mortality, treatment with telmisartan or irbesartan significantly suppressed aortic expansion in ApoE^−/−^ mice from days 3–28 and 7–28, respectively, following Ang II infusion as compared to mice maintained on standard chow (Table 1). Although mean aortic diameters on days 14, 21 and 28 in fluvastatin-treated, Ang II-infused mice were smaller than those in mice maintained on standard chow, these differences did not reach statistical significance. Doxycycline treatment also did not influence expansion of aortic diameter. Thus, in addition to reducing AAA incidence and mortality, telmisartan and irbesartan effectively suppress early aneurysm progression in the Ang II/ApoE^−/−^ model.

**Table 1:** Effect of telmisartan, irbesartan, fluvastatin and doxycycline on aortic diameters of Ang II-infused ApoE^−/−^ mice.

Treatment	Measurement Day						
	−7	0	3	7	14	21	28
Control	1.14±0.10 (15)	1.20±0.04 (15)	1.32±0.19 (15)	1.53±0.30 (12)	1.68±0.52 (12)	1.69±0.55 (10)	1.94±0.44 (10)
Telmisartan	1.13±0.10 (14)	1.13±0.06 (14)	1.08±0.07* (14)	1.10±0.07** (14)	1.12±0.05* (14)	1.11±0.10* (14)	1.14±0.05* (14)
Irbesartan	1.15±0.07 (14)	1.21±0.08 (14)	1.20±0.08 (14)	1.22±0.04** (14)	1.24±0.23** (14)	1.20±0.09** (13)	1.22±0.11** (13)
Fluvastatin	1.16±0.07 (14)	1.19±0.05 (14)	1.25±0.11 (12)	1.37±0.22 (11)	1.30±0.40 (10)	1.40±0.38 (10)	1.52±0.51 (10)
Doxycycline	1.13±0.07 (15)	1.17±0.10 (15)	1.20±0.10 (15)	1.39±0.28 (14)	1.64±0.44 (12)	1.95±0.52 (10)	2.05±0.55 (10)

Noninvasive transabdominal ultrasonography was used to measure suprarenal aortic diameters in individual mice prior to drug treatment (day -7), on the Ang II infusion day (day 0), and 3, 7, 14, 21 and 28 days after Ang II infusion. Data are given as mean ± standard derivation of the aortic diameters for all groups, with all dimensions listed in units of mm. Number of mice in each group is shown in the parenthesis. Two-way ANOVA analysis followed by Newman-Keuls post-test, *P<0.05 and **P<0.01 compared to the control group at same measurement day.

### ARBs Reduce Medial Elastolysis, Smooth Muscle Cell (SMC) Depletion and Transmural Aortic Inflammation

**Figure 2:**
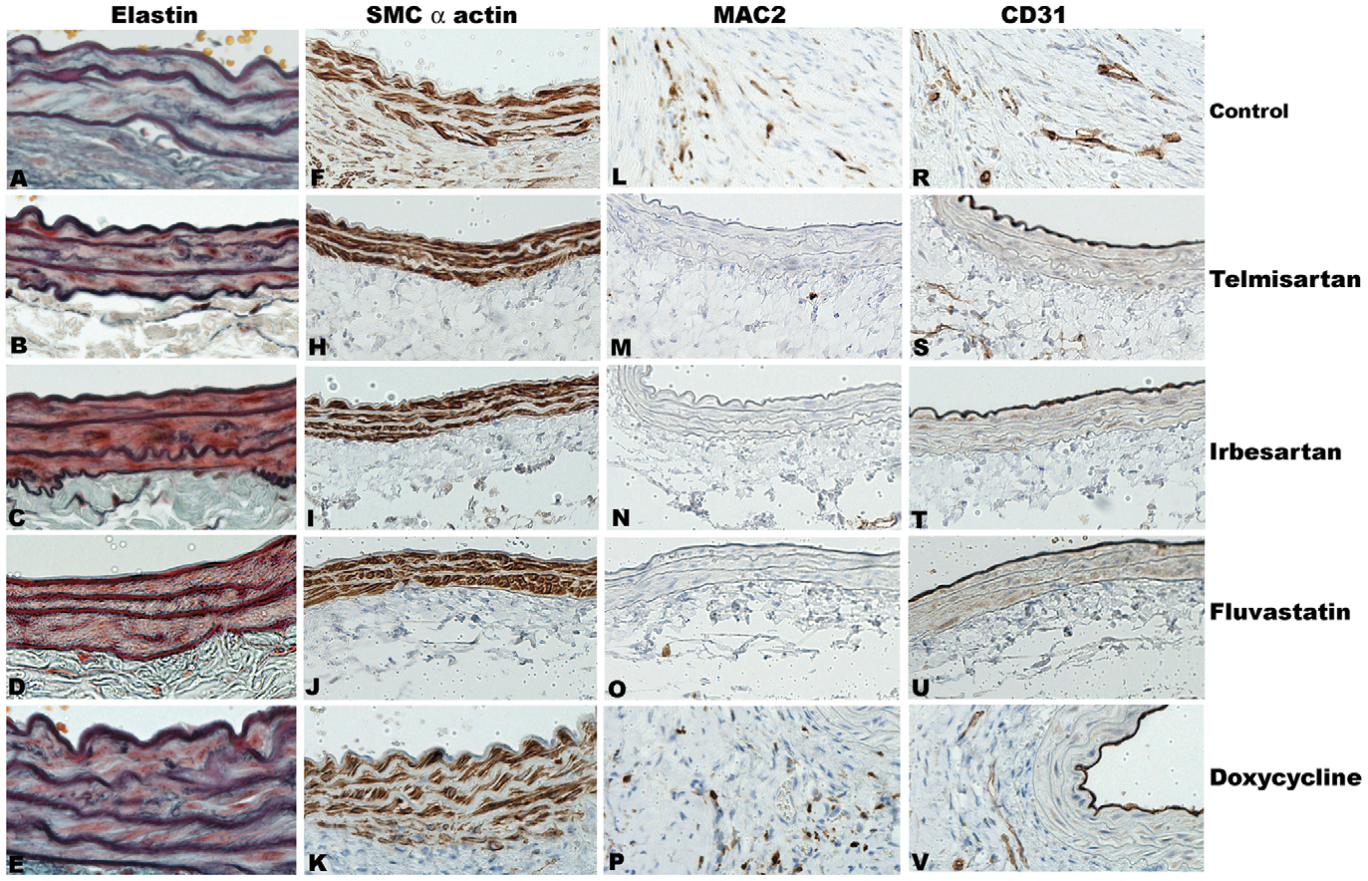
Influence of drug treatment on aortic histology. After sacrifice, aortae were fixed, sectioned and underwent elastin staining and immunostaining for SMCs (SMC α-actin), macrophages (MAC2) and blood vessels (CD31). **A–K:** Residual medial elastin (A–E) and mural SMCs (F–K) in telmisartan (B, H), irbesartan (C, I), fluvastatin (D, J), doxycycline-treated (E, K) or control mice (A, F). **L–V:** macrophages (L–P) and endothelial cells (neovessels) (R–V) in telmisartan- (M, S), irbesartan- (N, T), fluvastatin- (O, U) or doxycycline- (P, V) treated mice or untreated control mice (L, R). Representative images, 4–5 mice in each group. Magnification: x200 in A–E, x100 in F–V.

**Figure 3:**
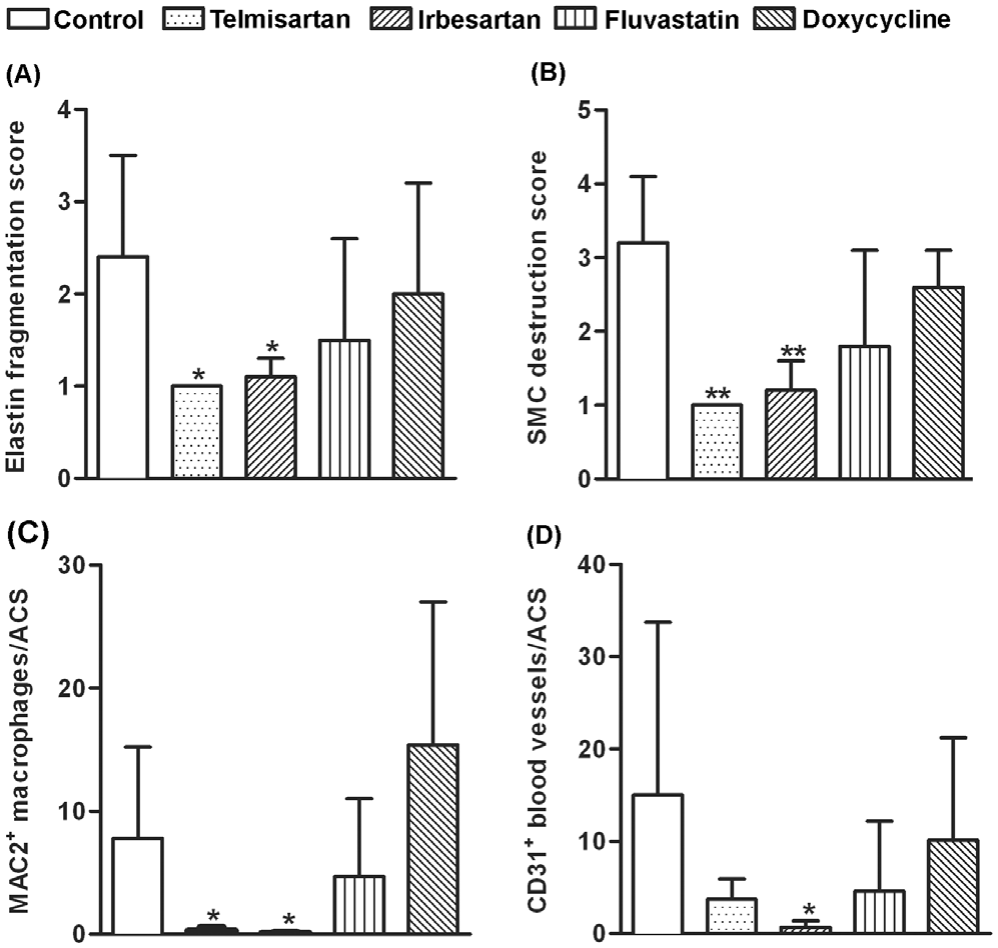
Quantitative aortic histology. Medial elastin fragmentation and SMC loss were assessed on a 1–4 scale. **A, B:** Destruction scores for medial elastin (A) and SMCs (B). **C, D:** Mural macrophages (C) and CD31^+^ neovessels (D) per aortic cross section (ACS). Data in A–D reported were mean±standard deviation, 4–5 mice per group. Nonparametric Mann-Whitney test, *P<0.05 and **P<0.01 compared to control group.

To determine the histopathological consequences of telmisartan or irbesartan treatment during aneurysm progression, we performed elastin and SMC α-actin staining on aortic sections from Ang II-infused ApoE^−/−^ mice and graded them as previously described [31]. Treatment with each ARB preserved medial elastin fibers and SMCs (Figs. 2A, B, C, D, E, F, G, H, I, J, K) compared to standard chow diet. In contrast, treatment with fluvastatin or doxycycline had no apparent effect on elastic fiber degradation or SMC depletion (Figs. 2 A, B, C, D, E, F, G, H, I, J, K, 3A & 3B).

Tissue immunostaining was used to evaluate the presence and magnitudes of monocyte/macrophage infiltration (MAC2 mAb) and newly formed blood vessels (mural angiogenesis, CD31 mAb) in AAAs. Treatment with either ARB reduced, to a remarkable extent, both mural macrophage and neovessel density within aneurysmal aortae compared to standard chow group (Figs. 2L, M, N, 2R, S, T & 3C). Neither doxycycline nor fluvastatin measurably influenced these two endpoints (Figs. 2L, 2O, 2P, 2R, 2U, V & 3C, D).

### ARBs Inhibit Expression of Proinflammatory Mediators and Proteinases

**Figure 4:**
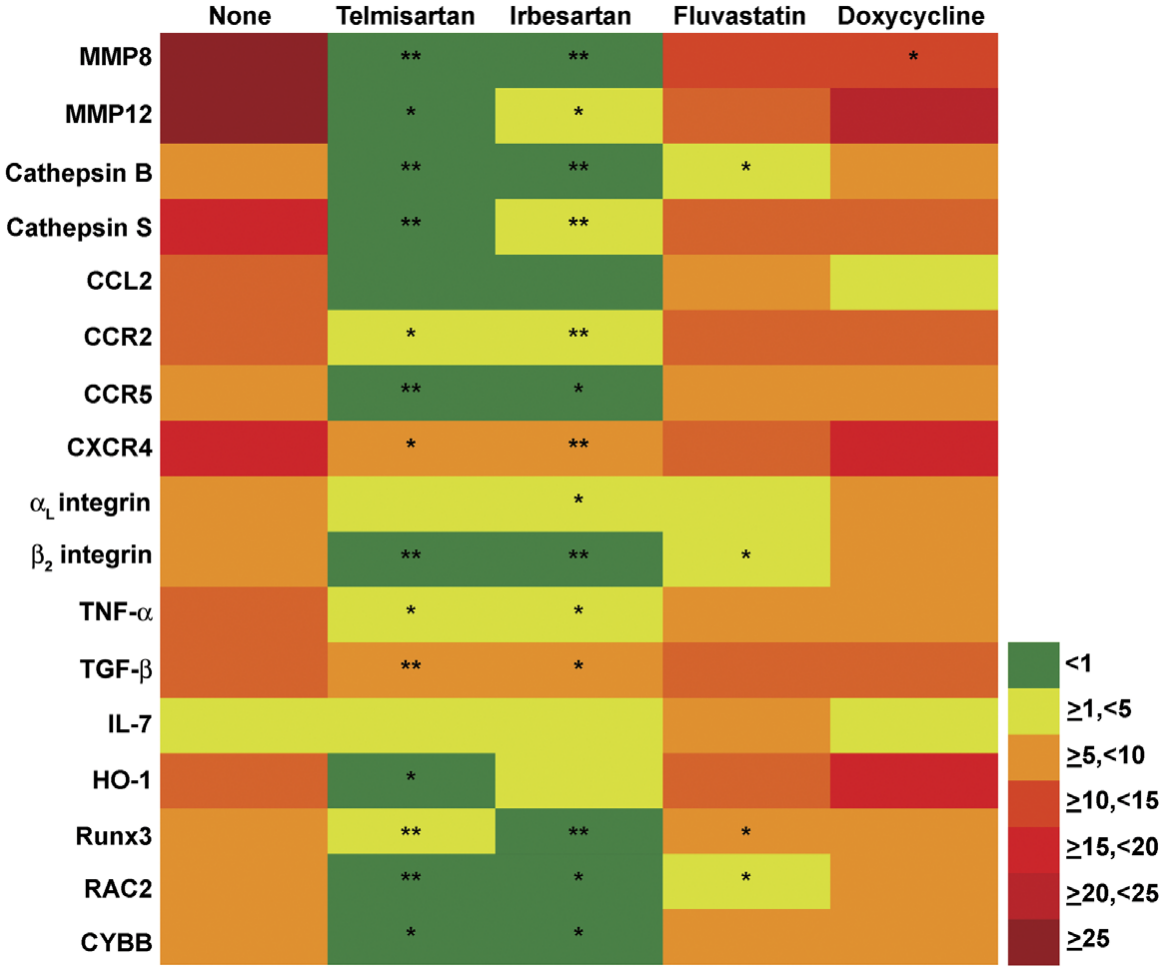
Influence of drug treatment on inflammatory gene expression. Aortic mRNA expression levels (fold expression) as a function of Ang II infusion (left column) and drug treatment status. Nonparametric Mann-Whiteny test, *P<0.05 and **P<0.01.

To further investigate the molecular mechanisms by which ARBs modify aneurysm pathogenesis, mRNA expression levels of 72 relevant inflammatory mediators were examined by quantitative real-time reverse transcription-polymerase chain reaction (qRT-PCR) (Table S1). These mediators were selected based on previous studies on gene expression in aneurysmal and occlusive aortic diseases. This transcription profile covered genes responsible for all facets of AAA pathology, including inflammation/immune responses, immunity, cell migration, cell proliferation and apoptosis, extracellular matrix metabolism, oxidative stress and cell signaling. As compared to aortae harvested from ApoE^−/−^ mice without Ang II infusion, mRNA expression levels of 19 genes were significantly increased in the aneurysmal aortae of the Ang II-infused mice, including five chemokines/chemokine receptors, three cytokines/cytokine receptors, four proteases, four anti-oxidative stress-related molecules, two leukocyte adhesion molecules and Runx3 (Fig. 4). The mRNA expression levels of 6 genes were significantly reduced, including tissue inhibitor of MMP2, fibronectin 1, colony stimulating factor 1, lysyl oxidase, NF-κB, and mitogen-activated protein kinase 8.

Treatment with either ARB significantly suppressed the Ang II-induced expression of MMP8, MMP12, cathepsin B, cathepsin S, chemokine CCL2, chemokine receptors (CCR2, CCR5 and CXCR4), α_L_ integrin, β_2_ integrin, TNF-α, TGF-β1, heme oxygenase 1, RAC2, CYBB and Runx3. Fluvastatin significantly downregulated expression of a smaller set of Ang II-induced genes, including cathepsin B, β_2_ integrin, RAC2 and Runx3, while doxycycline suppressed only MMP8. Interestingly, no drug treatment restored mRNA expression levels of genes significantly downregulated in Ang II-infused mice. Thus, ARBs suppressed expression of a far larger set of proinflammatory genes than either fluvastatin or doxycycline.

### Role of Blood Pressure Reduction in AAA Suppression

**Figure 5:**
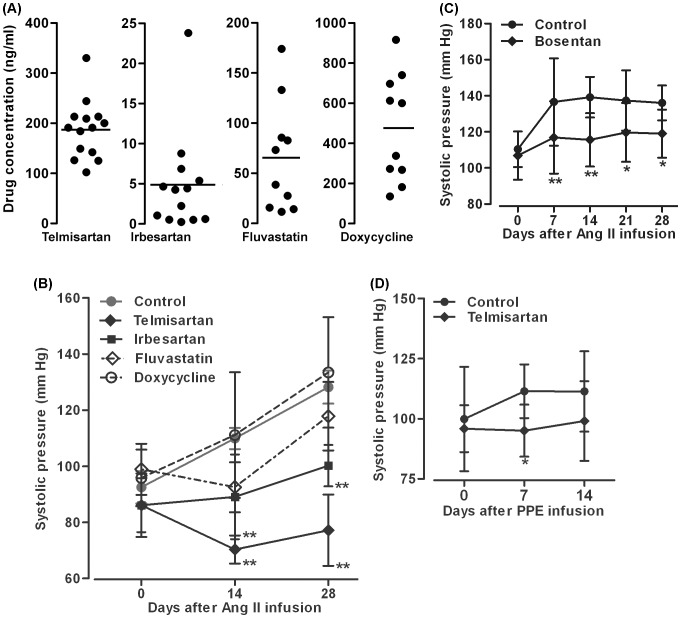
Effect of drug treatment on systolic blood pressure. **A:** Dot plot graphs show plasma drug levels in individual Ang II-infused ApoE^−/−^ mice receiving each drug treatment. Horizontal line in each graph indicates mean drug concentration from 10–14 mice in each group. **B:** Effect of telmisartan, irbesartan, doxycycline and fluvastatin on systolic blood pressure in ApoE^−/−^ mice 14 and 28 days after Ang II infusion. **C:** Effect of bosentan on systolic blood pressure in ApoE^−/−^ mice 7, 14, 21 and 28 days after Ang II infusion. **D:** Effect of telmisartan on systolic blood pressure in C57BL/6J mice 7 and 14 days after PPE infusion. Data in B–D are given as mean ± standard derivation for each group. Data in B and C were obtained from separate experiments, each with its own control group. In all experiments, two-way ANOVA followed by Newman-Keuls post-test, *P<0.05 or **P<0.01 compared to control group at same time points. n = 10–15 (A–C) or 7–9 (D) mice in each group.

**Figure 6:**
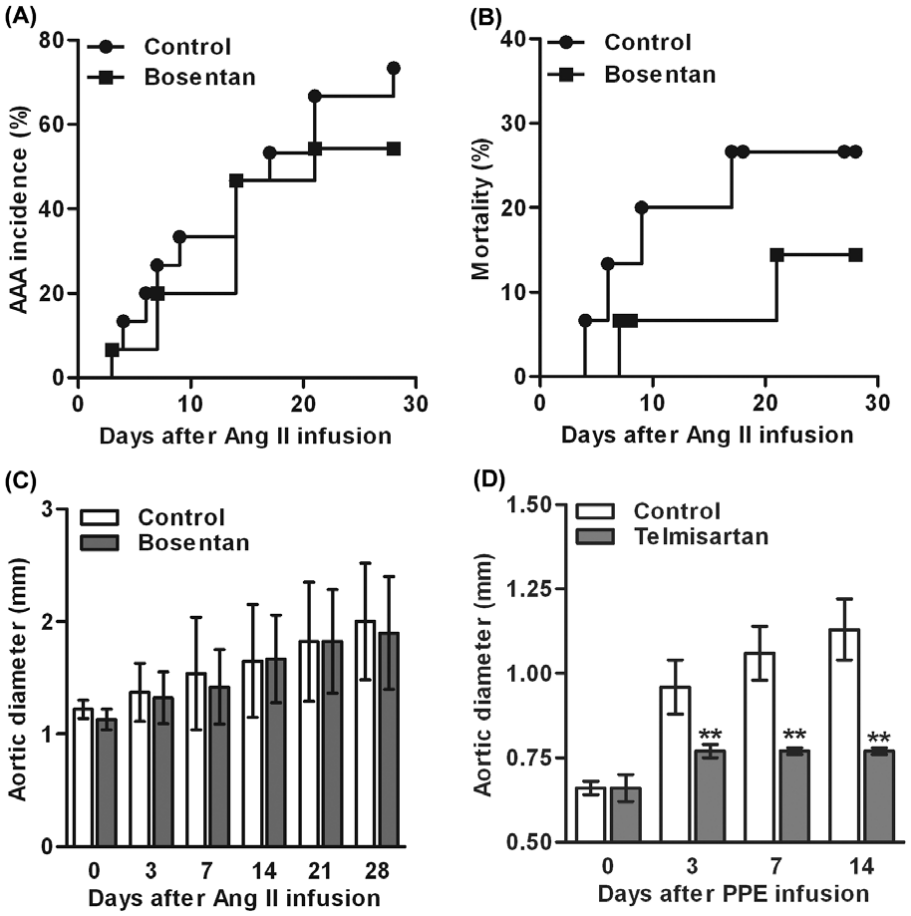
Antihypertension-independent effect of telmisartan on AAAs. A–C: Effect of bosentan on AAA incidence (A), mortality (B) and suprarenal aortic diameters (C) in Ang II-infused ApoE^−/−^ mice. **D:** Effect of telmisartan on infrarenal aortic diameters in C57BL/6J mice after PPE infusion. Data in B and D are given as mean ± standard derivation for each group. Two-way ANOVA followed by Newman-Keuls post-test, *P<0.05 or **P<0.01 compared to the control group at corresponding time points. n = 10–15 (A–C) or 7–9 (D) mice in each group.

Steady state plasma levels of telmisartan, irbesartan, fluvastatin and doxycycline are detailed in Fig. 5A. Drug levels were more consistent in ARBs vs fluvastatin or doxycycline-treated mice, although standard dosing methodology was employed across all groups. To determine the effect of individual drugs on Ang II-induced hypertension (a potential contributor to the formation and progression of AAAs), systolic blood pressure (SBP) was measured prior to Ang II infusion and at multiple time points thereafter. As shown in Fig. 5B, SBP was comparable between all four groups immediately prior to initiation of Ang II infusion. Subsequently, SBP was significantly lower in mice treated with telmisartan or irbesartan on days 14 and 28 of Ang II infusion. Neither fluvastatin nor doxycycline treatment influenced blood pressure. To determine whether blood pressure modulation account for some/all of the potent suppressive effect of ARB therapy on AAA development, we performed separate experiments in which Ang II/ApoE^−/−^mice were fed either with the chow supplemented with bosentan, a competitive endothelin-1 receptor antagonist and potent antihypertensive drug with no known influence on AT1a, or with standard chow as its own control. While bosentan was effective in blunting the Ang II-induced increase in SBP (Fig. 5C), it had no effect on AAA incidence or progression as compared to its own control group (Figs. 6A & 6C). Though mortality was slightly reduced in bosentan-treated Ang II/ApoE^−/−^ mice, this difference did not reach statistical significance compared to standard chow-fed control group (Fig. 6B). These experiments suggest that the AAA suppression by ARB therapy is not attributable in a significant way to blood pressure reduction alone.

### Telmisartan Suppresses Experimental AAA Formation in a Model-independent Manner

Finally, to determine whether ARB effectiveness was limited to Ang II-induced aneurysms, we tested the efficacy of telmisartan in a second murine AAA model, in which aortic aneurysmal degeneration is initiated via intra-aortic infusion of porcine pancreatic elastase (PPE) in normolipidemic C57BL/6J mice, without exposure to exogenous Ang II. This experimental construct also provided an additional control for the blood pressure-modulating influences of ARB therapy, since PPE does not induce hypertension of the magnitude or duration associated with Ang II infusion. Telmisartan alone was tested as the representative ARB in these experiments due to the larger overall aneurysm inhibitory effect demonstrated in the Ang II/ApoE^−/−^ model (Fig. 1). Treatment with telmisartan (10 mg/kg/d) almost completely obliterated aneurysmal aortic degeneration at 3, 7 and 14 days after PPE infusion (Fig. 6D). Within 2 weeks after PPE infusion, all mice fed with standard chow developed AAAs, whereas none of the mice fed with telmisartan-supplemented chow developed an AAA. Infusion of PPE increased SBP in a time-dependent manner (Fig. 5D). Although telmisartan treatment lowered SBP at 7 and 14 days, a significant difference was noted only at day 7. These results indicate that the potent inhibitory effect of ARB treatment in experimental AAA disease is model-independent and not solely the consequence of direct inhibition of Ang II/AT1a receptor interaction in hyperlipidemic mice.

## Discussion

These results demonstrated that both telmisartan and irbesartan are highly effective in reducing the incidence, progression and mortality of AAAs in the Ang II/ApoE^−/−^ model. Histologically, treatment with ARBs significantly reduced medial elastolysis and SMC loss. When multiple potential inhibitory agents were compared, ARBs demonstrated substantially more inhibitory activity than fluvastatin or doxycycline. As a control for ARB-induced blood pressure modulation, bosentan therapy alone did not influence aneurysmal aortic degeneration. Finally, telmisartan suppressed aneurysm formation in a second AAA model without exogenous Ang II administration, underscoring the potential translational relevance of this inhibition strategy.

These results validate and extend prior reports of AT1a inhibition in experimental and clinical AAA disease. An alternative ARB, losartan, inhibited AAA formation in the Ang II/ApoE^−/−^ model as evaluated by size and structural determination at sacrifice [13], and both ARBs telmisartan and valsartan limited PPE-induced aneurysm progression in rats [14], [15]. The pathophysiology and phenotype of AAAs are distinct from thoracic aneurysms, as associated with the Marfan syndrome, where extensive work by Dietz and others has established mechanisms likely responsible for ARB-mediated disease suppression [32]. This study supplements prior reports by evaluating ARB influences on aneurysm incidence, progression, and AAA-related mortality, all endpoints essential to potential translational considerations. Additionally, telmisartan was proved to be effective in distinct but complementary murine AAA models. Taken together with prior results, these data strengthen the accumulating evidence supporting ARB-based treatment regimens for human AAA disease management.

Clinical evidence supporting ARB efficacy in this application is less consistent. In a review of concurrent medication administered to AAA patients during serial ultrasound surveillance, ARB usage was associated with reduced AAA enlargement [16]. However, a prior population-based, retrospective case-control study conducted in the Provence of Ontario failed to identify a protective effect for ARB usage in reducing hospitalization for aortic rupture, although the number of AAA patients taking ARBs was small (less than 1% of population under review) [18]. Due to their retrospective nature, neither of these studies was able to control for relevant confounding variables that might independently influence AAA progression such as cigarette smoking or family history. To date, no prospective, randomized controlled trial has been conducted to evaluate the efficacy of ARB therapy.

This study provides additional mechanistic insights into ARB-mediated AAA suppression. Mural monocytes/macrophages are prevalent in human AAA tissue, and may mediate aneurysmal degeneration via expression of matrix-degrading proteinases, inflammatory cytokines, chemokines, and leukotrienes throughout the course of the disease [9], [22], [33]–[40]. Alternative macrophage populations such as those express TGF-β1 or heme oxygenase 1 may inhibit constitutive aortic cell loss and proteolysis, or contribute to positive aortic remodeling [36], [41], [42]. Mechanisms responsible for reduced aortic macrophage density in ARB-treated mice may include impaired monocyte mobilization from spleen and/or bone marrow, impaired aortic localization and transmural migration, and/or reduced macrophage survival *in situ* [43]–[46].

Mural angiogenesis is another well-recognized pathologic component of AAAs, recapitulated in murine AAA models, and correlates with disease progression in experimental models and human disease [47]–[51]. Previous work employing loss- and gain-of-function experimental strategies has highlighted the significance of angiogenesis in AAA pathogenesis [49]–[55]. Angiogenesis may also facilitate compensatory aortic remodeling in the diseased aorta [50], [56]. The proangiogenic cytokine VEGF-A is produced by inflammatory monocytes/macrophages [47], [57], [58], thus ARB treatment may inhibit angiogenesis through limitations on macrophage accumulation. Alternatively, AT1 receptor inhibition may exert direct antiangiogenic influences as reported for other pathologic conditions [59]–[61]. Irrespective of the specific mechanisms responsible for this inhibitory effect, reduced mural neovessel formation limits further recruitment of circulating monocytes, amplifying the suppressive effect on aneurysm progression.

Analysis of ARB-induced changes in proinflammatory gene expression provides additional insights into potential antianeurysmal effects. Dramatic reduction in CCL2, CCR2, CCR5, CXCR4, α_L_ and β_2_ integrins were noted, all genes highly expressed in the aneurysmal aortae. Monoclonal antibody inhibition or genetic deficiency of CCL2, its receptor CCR2, or CD18 has previously been shown to ameliorate experimental AAAs [35], [37], [62]. Recently, we demonstrated that synthetic peptide inhibition of CCL5 (a major CCR5 ligand) or genetic deficiency of ICAM-1 also suppresses PPE infusion-induced AAAs in mice (unpublished data). Similarly, aortic monocyte/macrophage attenuation by ARBs may reduce CCL2 production either directly or via interaction with aortic SMCs [63]–[65].

Gene expression levels of MMP8, MMP12, cathepsin B, cathepsin S, TNF-α and OPN, all highly differentially expressed in aneurysmal aortae, were significantly downregulated in ARB-treated mice. These enzymes and mediators all contribute to AAA formation by aortic extracellular matrix degradation, proangiogenic or proinflammatory signaling [33], [36], [39], [66]. Differential expression may reflect reduced mural monocyte/macrophage infiltration and/or phenotypic switch towards alternatively activated antiinflammatory macrophages [67], [68]. Although tissue inhibitor of MMP2, lysyl oxidase and fibronectin 1, enzymes and structural proteins thought to inhibit proteinase activity or stabilize the extracellular matrix [69]–[74], were downregulated in aneurysmal lesions, reciprocal upregulation was not apparent in ARB-treated mice despite the suppressive effects on aneurysm incidence and progression. Taken together, the expression data provides further support to the hypothesis that ARBs suppress experimental aneurysm formation by limiting activity of proinflammatory macrophages. By down-regulating expression of aortic CCL2, CCR2, CCR5, CXCR4 and α_L_ and β_2_ integrin expression, ARBs significantly limit the adhesion and transendothelial aortic migration of circulating monocytes, differentiation into a proinflammatory phenotype, and the production of inflammatory cytokines and proteinases.

Several limitations to our gene expression analysis exist. First, mRNA expression levels of TGF-β1 and heme oxygenase 1 were not consistent with prior reports of compensatory upregulation in response to aneurysm formation [36], [41], [42], although their specific roles in AAA pathogenesis remain controversial. Second, expression of MMP-9, which plays a critical role in aneurysmal pathogenesis [22], was apparently not influenced by ARB treatment, although earlier expression in the time-course of experimental AAA formation, when MMP-9 activity may be more essential, was not assessed. Third, mRNA expression levels of Runx3, RAC2 and CYBB were also influence by ARB treatment. Although they are likely involved in AAA pathogenesis through their roles in the generation of reactive oxygen and nitrogen species, angiogenesis and myeloid cell functions [75]–[77], their specific contributions could not be identified in this experimental construct. Fourth, aortae were harvested for RNA extraction following 28 days of Ang II infusion, representing late stage disease, with mixed populations of infiltrative and constitutive cells. Thus, this experimental design precluded analysis of cell- or time-dependent gene expression. Finally, Ang II binds to either AT1 or AT2 receptor on target cells, resulting in opposite biological consequences. Whether exogenous Ang II downregulates AT1 mRNA levels and/or leads to compensatory upregulation of AT2 mRNA levels was not evaluable within this study design. AT2 signaling may be essential for deriving the maximal therapeutic benefits of ARB-treated murine Marfan syndrome models [78]. Further studies will be required to document the suppressive role of AT2 expression and signaling in AAA models.

Prior experiments suggested that endothelial AT1a receptor expression and activity, but not hematopoietic or vascular smooth muscle cell expression, is required for Ang II-induced AAA formation in ApoE^−/−^ mice [10], [11]. However, differentiation of monocyte lineage progenitors from hematopoietic stems cells is regulated by AT1a-mediated TNF-α secretion by bone marrow stromal cells [79]. Monocyte AT1a activation is critically important for egress of splenic monocytes into the bloodstream in certain pathological conditions including myocardial infarction [43]. Thus, more studies are needed to define the comprehensive consequences of Ang II/AT1a signaling in experimental and clinical AAA disease.

The potential influence of ARB-induced blood pressure reduction on aneurysm suppression was investigated using two complementary approaches. First, telmisartan efficacy was confirmed in the PPE infusion model to negate the effect of Ang II-induced hypertension on aneurysm progression. Second, blood pressure reduction with bosentan alone had no influence on AAA initiation and progression in the AngII/ApoE^−/−^ model. Recent data suggests that bosentan may augment aneurysm incidence in Ang II-infused ApoE^−/−^ mice [80], although our data did not support this conclusion. Therefore, AAA suppression in these constructs did not appear to be blood pressure-dependent, although we did not test doses below the therapeutic threshold for blood pressure modulation. Telmisartan also has well-documented PPAR-γ agonist activity [19], [81], and activation of PPAR-γ has proven effective in limiting experimental aneurysm progression [31], [82], [83]. Determining the relative contribution of the PPAR-γ agonist vs Ang II/AT1 antagonist effects of telmisartan in limiting experimental aneurysm progression, while of significant scientific interest, is beyond the scope of this investigation. Further planned studies will compare the independent influences of PPAR-γ and AT1 signaling on AAA progression in these models.

Reflecting the existing ambiguity regarding the efficacy of these agents in the literature [20], [27], [29], [84]–[91], we found that both fluvastatin and doxycycline demonstrated little or no effect on aneurysm formation or progression in Ang II-infused ApoE^−/−^ mice. The clinical relevance of these findings in the case of fluvastatin was supported by a recent meta-analysis demonstrated no significant effect of statins on clinical AAA progression, although a significant reduction in all-cause mortality was noted [25], [26]. In the case of doxycycline, while no definitive clinical trial data have been published to date [25], [26], multiple prior reports have demonstrated its effectiveness in limiting PPE-induced experimental AAAs in both mice and rats [21]–[23]. In the Ang II/ApoE^−/−^ model, however, published results have varied significantly [20], [24], [92]. One of these studies reported a 50% reduction in AAA incidence in Ang II-infused, doxycycline-treated ApoE^−/−^ mice fed with high fat chow [24], suggesting that dietary fat intake may influence the therapeutic effect of doxycycline in this model. Since the doxycycline dose employed in our study is at the high end of prior published regimens shown to suppress AAAs in the PPE model [21], it is thus unlikely that failure could be ascribed to an insufficient dose. Thus, these results add to the uncertainty surrounding the therapeutic efficacy of statins and doxycycline in AAA disease.

In conclusion, our study proved that telmisartan and irbesartan, but not doxycycline, fluvastatin or bosentan, are highly efficacious in suppressing the formation and progression of AAAs in a model- and blood pressure-independent manner. RAS inhibition with ARBs may represent an attractive pharmaceutical strategy for suppression of early AAA disease. If proven effective, ARB therapy may prolong the time to surgical repair for many patients, and in the most elderly, potentially provide the opportunity to forgo surgery altogether [3]. On the basis of these experiments and additional observational clinical data, a randomized, double-blind, placebo-controlled, multiple center clinical trial has been organized to test the efficacy of telmisartan in limiting the progression of early abdominal aortic aneurysm disease. As one of several centers participating in this international trial, we are treating AAA patients with aneurysm diameters between 35–49 mm by daily administration of 40 mg telmisartan vs placebo for up to 2 years (www.clinicaltrials.gov). If proven effective clinically, this strategy promises to substantially improve patient well-being and quality of life for thousands of “worried well” patients world-wide at risk for progressive aneurysm enlargement and sudden death due to aortic rupture.

## Materials and Methods

### Aneurysm Creation and its Intervention

Male ApoE^−/−/^C57BL/6J mice or wild type C57BL/6J mice at 10–12 wk of age were obtained from the Jackson Laboratory, Bar Harbor, Maine, and housed at the Stanford Animal Facility, Stanford, CA. Animal care and experimental procedures were conducted in compliance with Stanford Laboratory Animal Care Guidelines. The Administrative Panel on Laboratory Animal Care at Stanford University approved all procedures involving mice.

Two mechanistically distinct, but complementary mouse AAA models were used in this study: subcutaneous Ang II infusion in ApoE^−/−^ mice (Ang II/ApoE^−/−^ model) and intra-aortic PPE infusion in C57BL/6J mice (PPE model). In most experiments, ApoE^−/−^ mice were fed chow supplemented with irbesartan (50 mg/kg), telmisartan (10 mg/kg) or bosentan (100 mg/kg), or were daily given drinking water supplemented with fluvastatin (40 mg/kg) or doxycycline (100 mg/kg). As controls, separate groups of ApoE^−/−^ mice for individual experiments were given the standard chow and drinking water without drug supplementation. One week later, to induce AAAs, all mice were subcutaneously implanted with osmotic minipumps (Alzet model 2004, Durect Corporation, Cupertino, CA) for continuous infusion of Ang II at 1000 ng/kg/min, and treated continuously with their respective drugs for 28 days [9]. In additional experiments, C57BL/6J mice were fed telmisartan-supplemented chow (10 mg/kg) or the standard chow. One week thereafter, AAAs were created by transient intra-aortic infusion of PPE as described previously [93], and these mice were continuously fed with the chow with or without telmisartan supplementation for additional 2 wk. In all experiments, doses for two ARBs and bosentan were selected based on published mouse studies in which each drug lowered blood pressure and/or suppressed cardiovascular pathology [94]–[96].

### Measurements of Plasma Drug Concentrations

Plasma samples were obtained from the ApoE^−/−^ mice at sacrifice following 28 days of continuous Ang II infusion. Drug levels in plasma were measured using high pressure liquid chromatography and presented as ng/ml.

### Measurements of Blood Pressure

Systolic blood pressure was measured in conscious, pre-warmed (36°C) and restrained mice using a noninvasive tail-cuff method on a BP-2000 system (Visitech Systems, Inc., Napa Place Apex, NC). Measurements were performed prior to Ang II infusion (day 0) as well as 14 and 28 days thereafter. At each measurement day, a total of 15 measurements for each mouse were conducted with a 5 second interval.

### Monitoring of AAA Formation Using Transabdominal Ultrasonography

Aortic diameter measurements and dissection-flap recognition were obtained via ultrasound imaging at 40 MHz using the Vevo 770 ultrasound system (Visualsonics, Toronto) in a blind manner for each medication group. All ultrasound measurements of aortic diameters were performed by a single investigator and had an inter-measurement variation of less than 2%. Imaging was performed prior to Ang II infusion and on days 3, 7, 14, 21 and 28 thereafter. An AAA was defined as a ≥50% increase in aortic diameter or the presence of aortic dissection. Mice were daily monitored for mortality analysis. Dead mice were subjected to necropsy within 12 h to confirm presence or absence of aortic rupture.

### Histological Analysis

Mice were sacrificed following 28 days of Ang II infusion or on day 14 following PPE infusion. Aortae were harvested, fixed with 4% PFA in phosphate-buffered saline (PBS), embedded in paraffin, and sectioned (4 µ m in thickness). Elastic-Masson staining was used to stain medial elastin. Immunohistochemistry was used to identify SMCs, macrophages and blood vessels. In brief, the PBS-rehydrated sections were incubated with a rabbit anti-mouse SMC α-actin polyclonal antibody (Laboratory Vision, Fremont, CA), a rat anti-mouse MAC2 mAb (M3/38, Cedarlane Laboratories, Burlington, Ontario, Canada), a rabbit anti-mouse CD31 polyclonal antibody (Laboratory Vision, Fremont, CA), or a species and isotype-matched negative control antibody. Following extensive washing with PBS, the sections were sequentially incubated with an appropriate biotinylated secondary antibody and streptavidin-peroxidase. The binding of a primary antibody to its specific antigen was visualized using the DAB kit (Dako Corporation, Carpinteria, CA) and imaged on a light microscope equipped with a Nikon digital sight DS-5M camera using the NIS Elements software (Ver. 3, Nikon Instruments Inc, Melville, NY). Macrophages and blood vessels within the aortic wall were quantitated as MAC2^+^ cells and CD31^+^ vessels per aortic cross section (ACS), respectively. Destruction of medial elastin and smooth muscles was graded as 1 (mild) to 4 (severe) [31]. All histological assessments were performed by a single experienced experimental pathologist who was blinded to treatment assignments or groups.

### Quantitative Real-time Reverse-transcription Polymerase Chain Reaction (qRT-PCR)

Total aortic RNA was extracted using the Qiazol lysis reagent and RNeasy Lipid Tissue Mini kit (Qiagen Inc, Valencia, CA). After removal of genomic DNA by DNase 1 treatment, cDNA was synthesized from 1 µg total RNA using the Applied Biosystems high reverse cDNA transcription kit, and amplified on the ABI PRISM 7900HT Sequence Detection System using a 384-well Applied Biosystems Taqman low density array card (Foster City, CA). The total volume of PCR reaction was 100 µl, each containing cDNA synthesized from 50 ng total RNA and 50 µl Taqman universal PCR master mix. PCR was run by activating DNA polymerase at 50°C for 2 minutes and denaturing cDNA at 95°C for 10 minutes followed by 40 cycles of 2-step amplification (denaturation at 95°C for 15 seconds and annealing/extension at 60°C for 1 minutes). A house keeping gene, GAPDH, was used as an internal control for all qRT-PCR reactions. All data were analyzed using SDS 2.2.3 software (Applied Biosystems), and gene expression levels in all Ang II-infused groups were expressed as fold changes as compared to no Ang II-infused group in which each gene expression level was set at 1.0.

### Statistical Analysis

For all continuous variables, data were presented as mean and standard derivation (SD) unless indicated, and nonparametric Mann-Whitney test or two-way analysis of variance (ANOVA) followed by Newman-Keuls post-test was used to test for significance. The differences in the trends for AAA incidence and mortality between the groups were tested by Kaplan-Meier analysis. *P*<0.05 was considered to be statistically significant.

## Supporting Information

Table S1 (XLSX)
